# Plateau Adaptation Gene Analyses Reveal Transcriptomic, Proteomic, and Dual Omics Expression in the Lung Tissues of Tibetan and Yorkshire Pigs

**DOI:** 10.3390/ani12151919

**Published:** 2022-07-27

**Authors:** Peng Shang, Bo Zhang, Pan Li, Zulfiqar Ahmed, Xiaoxiang Hu, Yangzom Chamba, Hao Zhang

**Affiliations:** 1 Laboratory National Engineering for Animal Breeding, Department of Animal Genetics and Breeding, China Agricultural University, Beijing 100193, China; nemoshpmh@126.com (P.S.); bozhang0606@cau.edu.cn (B.Z.); ldpan528@163.com (P.L.); huxx@cau.edu.cn (X.H.); 2 Department of animal husbandry, College of Animal Science, Tibet Agriculture and Animal Husbandry College, Linzhi 860000, China; 3 Faculty of Veterinary and Animal Sciences, University of Poonch Rawalakot, Rawalakot 12350, Pakistan; zulfiqarahmed@upr.edu.pk

**Keywords:** plateau adaptation gene, transcriptomic, proteomic, Tibetan pigs, Yorkshire pigs

## Abstract

**Simple Summary:**

The low oxygen concentrations of high-altitude regions hinder their development possibilities. In this investigation, we used lung tissue from the adopted Yorkshire sow and from the Tibetan pig to analyze the occurrence and development mechanisms of high-altitude hypoxia using dual expression omics. Seven key candidate genes (*SELENBP1*, *MCC*, *CAPG*, *ASS1*, *ADH4*, *LYZ*, and *CPS1*) were screened from the lung tissues and found to be predominately involved in mitochondrial function, blood particle regulation, glycolysis, ethanol oxidation, and the Wnt signaling pathway, as well as other related hypoxia-adaptive regulatory mechanisms.

**Abstract:**

Elevated environments such as plateaus are often classified as low oxygen environments. The hypoxic adaptation mechanisms utilized by organisms in these conditions are not well understood. To address this, the differentially expressed genes (*DEGs*) involved in hypoxia adaptation were assessed using two pig breeds (Tibetan pig [TP] and Yorkshire sow [YY]). Genes related to lung tissue responses to hypoxia were assessed using transcriptomic (using RNA-seq) and proteomic (using iTRAQ) analysis. A total of 1021 DEGs were screened out. In the iTRAQ omics data, a total of 22,100 peptides were obtained and 4518 proteins were found after filtering. A total of 271 differentially expressed proteins [DEPs] were screened using the conditions of *p* < 0.05; FC ≤ 0.833; and FC ≥ 1.2. A total of 14 DEGs at the mRNA and protein levels were identified and found to be associated with regulation of the inflammatory response; blood particles; and MAPK cascade response regulation. Among the DEGs, six were associated with hypoxia adaptation function (mitochondria and glycolysis) in pigs. The results of this study identify novel candidate genes involved in porcine hypoxia adaptation mechanisms.

## 1. Introduction

Oxygen is required to sustain life for most living organisms [1]. However, the responses of different organisms to different oxygen environments are not uniform [2] and hypoxia adaptability has been identified as a complex mechanism involving multiple genes and regulatory networks. Understanding the effects of hypoxia to improve the adaptability and improvement of livestock hereditary traits, the introduction of better adopted individuals to high plateau environments, and the prevention and treatment of certain plateau diseases is required [3,4,5]. The occurrence and development of hypoxia adaptation mechanisms and the specific complex network regulation mechanism has not yet been explored [6]. The Tibet region of China is a natural plateau with a hypoxic environment [7]. Owing to the development of the Tibetan region, foreign pig breeds such as the Yorkshire sow (YY) have been introduced in recent years to fulfill nutritive requirements. Endogenous Tibetan pigs (TP) have good stability and genetic adaptability to the hypoxic environment of the plateau and consequently they should be studied to better understand plateau hypoxia adaptability [8,9,10].

The mechanisms of plateau hypoxia adaptation can be investigated using transcriptomic, proteomic, and dual omics analyses to study differentially expressed genes (DEGs) and differentially expressed proteins (DEPs) at different expression levels. In addition, RNA-seq data analysis has been used to help identify genes and pathways related to altitude hypoxia adaptability [11]. Isobaric tags for relative and absolute quantitation (iTRAQ) omics data have been used to explore batches of differentially expressed proteins related to the development of hypoxia and hypoxia adaptation mechanisms in tissues [12]. Integrated dual-omics analysis [13] used DEGs and DEPs screened by two-omics analysis to identify differences and common points in important candidate genes related to hypoxic adaptation mechanisms. In the present study, we used adopted YY and TP pigs to analyze the occurrence and development of mechanisms for high altitude hypoxia using dual expression omics of lung tissue.

## 2. Materials and Methods

### 2.1. Ethics Statement

In the present study, the experimental animals were domestic pigs that were not endangered or protected. The rearing, slaughtering, and experimental conditions strictly followed the guidelines approved by the Animal Welfare Committee of the State Key Laboratory of Agricultural Biotechnology of China Agricultural University (Approval number: XK257).

### 2.2. Animal and Sample Preparation

All experimental pigs were born and bred in the practice ranch of the Tibet Agriculture and Animal Husbandry College, Tibet Autonomous Region (elevation: 2900 m). All pigs were housed in standard conditions with natural, uncontrolled room temperature and light. Complete formula meal feed was fed three times per day and pigs had ad libitum access to water. At the age of 6 months, nine TP and nine YY were randomly selected to slaughter and sample according to the guidelines approved by the Animal Welfare Committee of the State Key Laboratory of Agricultural Biotechnology of China Agricultural University (Approval number: XK257). Approximately 5 mg of lung tissue was collected from each individual at the same site and placed in 2 mL cryopreservation tubes, with 2 tubes per individual, immediately frozen in liquid nitrogen, and stored at −80 °C for total RNA and total protein extract.

### 2.3. Total RNA and Protein Isolation from Lung Samples

Extraction of the total RNA from the lung tissues was performed using Trizol reagent (Invitrogen, Carlsbad, CA, USA). The purity of the RNA samples was tested with a Nanodrop 2000 microspectrophotometer (Thermo Fisher Scientific Inc., West Palm Beach, FL, USA). The concentration and integrity of the total RNA was investigated using a 2100 Bioanalyzer, RNA 6000 Nano Kit (Agilent, Carlsbad, CA, USA). The extraction of the total protein from the lung tissue was done using the RIPA cracking method (Beyotime Ltd., Shanghai, China). The quantification of protein was carried out using the BCA Protein Assay Kit (Beyotime Ltd., Shanghai, China) and integrity was determined using polyacrylamide gel electrophoresis.

### 2.4. Library Preparation and RNA Sequencing

Prior to sequencing, samples of the same species were randomized into groups of three. After enrichment and purification using magnetic beads with Oligo (dT), a cDNA library was added to the fragmentation buffer to generate short fragments that were used as a template. The first strand of the cDNA was synthesized with six base random primers and the second strand of cDNA was synthesized by adding buffer, dNTPs, RNaseH, and DNA polymerase I, purified using a QIAQuick PCR kit (QIAGEN, Hilden, Germany), and eluted with EB buffer. The purified double-stranded cDNA was eluted and then subjected to end repair, base A, and sequencing adapter treatment. Later, the target size fragments were obtained by agarose gel electrophoresis and PCR amplification to complete the entire library preparation.

After library construction, Qubit 3.0 (Life Technologies, Carlsbad, CA, USA) for preliminary quantification was performed. The library was diluted to 1 ng/μL, and then the Agilent 2100 (Agilent Technologies, Carlsbad, CA, USA) was used to detect the insert size of the library. After the expected insert size was obtained, Bio-RAD CFX 96 (Bio-RAD, Hercules, CA, USA) fluorescence quantitative PCR Bio-RAD Kit iQ SYBR GRN (Bio-RAD, Hercules, CA, USA) performed q-PCR to accurately quantify the effective concentration of the library (effective library concentration >10 nM) and to ensure the quality of the library. The qualified libraries were sequenced using the Illumina platform and the sequencing strategy was PE150. Illumina high-throughput sequencing was used for mapping and alignment of the sequence reads. The results that originally existed in the image data file were converted into sequenced reads using bcl2fastq, called raw data files. We obtained a clean, high-quality sequence by removing the low-quality sequence from the original sequence. Afterwards, HISAT2 was used to compare the obtained clean reads with the reference genome Sus scrofa 11.1 to map it to the genome. Data analysis was performed using SPSS Statistics 23 (International Business Machines Corporation, Armonk, NY, USA), and heatmaps were drawn using the R language pheatmap package (1.0.12) [14].

### 2.5. Differential Gene Analysis Using RNA-seq

RNA-seq analysis was performed by counting the number of sequences (reads) located in the genomic region or exon region and expressing them using FPKM (per million fragments) to obtain the number of fragments per kilobase length in a gene. Screening conditions |log2FoldChange| > 1 and *p* < 0.05 were used to identify DEGs. The functional annotation and pathway enrichment of the key differentially expressed genes was carried out using pathway analyses and literature studies. Correlations were later observed with the hypoxic adaptation mechanisms.

### 2.6. Proteolysis and Labelling 

The enzymatic digestion of the quantified protein was performed using FASP enzymatic technology. The iTRAQ Reagent-8Plex Multiplex kit (Sigma-Aldrich, St. Louis, MO, USA) was used to label the enzymatic product according to the manufacturer’s instructions. The protein samples were extracted from lung tissue and divided into three biological replicates, each including three individuals, labeled as 113 (TP1), 114 (TP2), 117 (TP3), 118 (YY1), 119 (YY2), and 121 (YY3). High performance liquid chromatography (HPLC) was used to classify the product on a C18 column under high pH conditions. Different gradients were setup to elute the product, which was then freeze-dried under vacuum and re-dissolved in 5 μL 0.5% FA. The flow rate was set to 700 nL/min and the eluent was A (98% H_2_O, pH10) and B (98% ACN, pH10). The 60 collected components were combined into multiple components for fractionation and each sample was separated using a nanoliter flow rate HPLC liquid system. The column was equilibrated with 95% solution A (0.1% FA, H_2_O). The sample was loaded from the autosampler to the pre-column of the mass spectrometer, and the peptide was separated using the analytical column. The flow rate was set to 600 nL/min, and the eluent was A (0.1% FA, H_2_O) and B (0.08% FA, 80%). After separation by capillary HPLC, a mass spectrometer Orbitrap Fusion™ Lumos™ Tribrid™ (Thermo Scientific™, Waltham, MA, USA) was used for mass spectrometry analysis. The detailed steps for enzymolysis and iTRAQ labeling have been described previously [15,16].

### 2.7. Database Search, Protein Identification, and Quantification

The database search software Proteome Discoverer was used to search the obtained data in the database Uniprot Sus_ = scrofa (downloaded from 2 January 2019, a total of 49,003 sequences). After filtering the obtained peptide data with a false discovery rate (FDR) ≤ 0.01, the peptides were identified. Quantitative analysis of the peak intensity values reported by peptides was performed. All identified proteins were screened under fold change ratio (FC) conditions of ≥1.2 or ≤0.83, and *p* ≤ 0.05. The online website was used to perform sysbal name conversion for the DEGs and DEPs (https://biodbnet-abcc.ncifcrf.gov/db/db2dbRes.php, accessed on 10 January 2020). GO analysis was performed on the genes after the name conversion and annotations of the KEGG pathway. The online software Metascape (http://metascape.org/gp/index.html#/main/step1, accessed on 10 January 2020) was used to classify and annotate the differential genes and differential proteins [17]. At this time, *p* < 0.05 was selected as the pathway with significant difference for subsequent analysis.

### 2.8. RT-qPCR of Candidate Genes

The analysis was combined with dual-omics and the candidate genes screened. Real-time fluorescence quantification methods were used to analyze gene mRNA expression to determine whether the data from gene-specific expression patterns were reliable.

## 3. Results

### 3.1. Summary of RNA-seq Data

**Figure 1:**
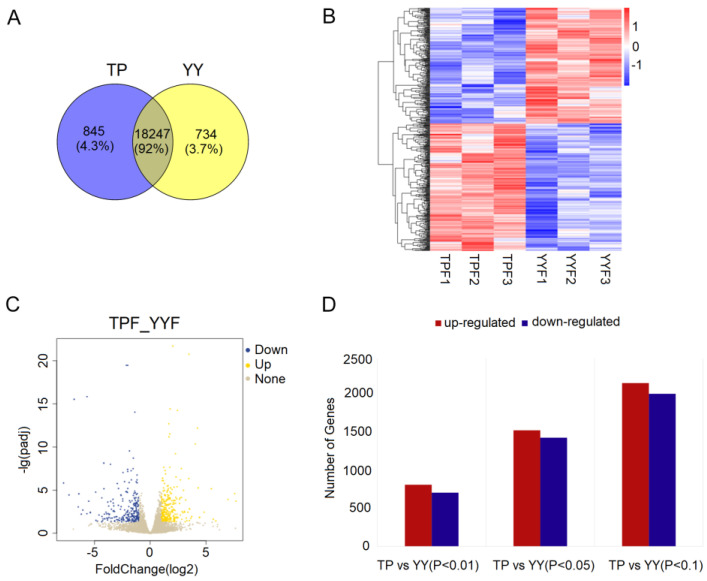
Cluster analysis of differentially expressed genes. Venn diagram of the number of genes expressed in each group (**A**); heatmap of differentially expressed genes between the two groups (**B**); volcano plot of differentially expressed genes (**C**); and differentially expressed genes (**D**). YY, Yorkshire pig; TP, Tibetan pig.

After removing the linker and low-quality sequences, each sample obtained 44.6–48.0 million clean reads; approximately 95% of the clean reads were mapped to the Sus scrofa genome sequence. Taking the calculated FPKM value as the gene expression level, a total of 19,826 expressed genes were observed in the lung tissue. Of the expressed genes, 18,247 were co-expressed between the two groups (Figure 1A). A heat map of all co-expressed genes showed the biological repeatability within each group, with differences in the transcriptome patterns of the TP and YY (where red indicates a high expressed gene and blue indicates a low expressed gene; Figure 1B). A volcano plot was used to clearly reflect the obviously expressed unigenes using yellow and blue for the two groups. The results showed that 1012 genes were up-regulated and 980 were down-regulated in the TP groups when compared with those in the YY groups (Figure 1C). The top 20 significantly different up-regulated and down-regulated genes in the two groups are listed in Table 1. There were approximately 4200 (*p* < 0.1), 2988 (*p* < 0.05), and 1534 (*p* < 0.01) significant DEGs identified between the TP and YY groups, including up-regulated (2171, 1543, and 820) and down-regulated (2029, 1445, and 714) UniGene IDs (Figure 1D).

### 3.2. Functional Annotation of DEGs

**Figure 2:**
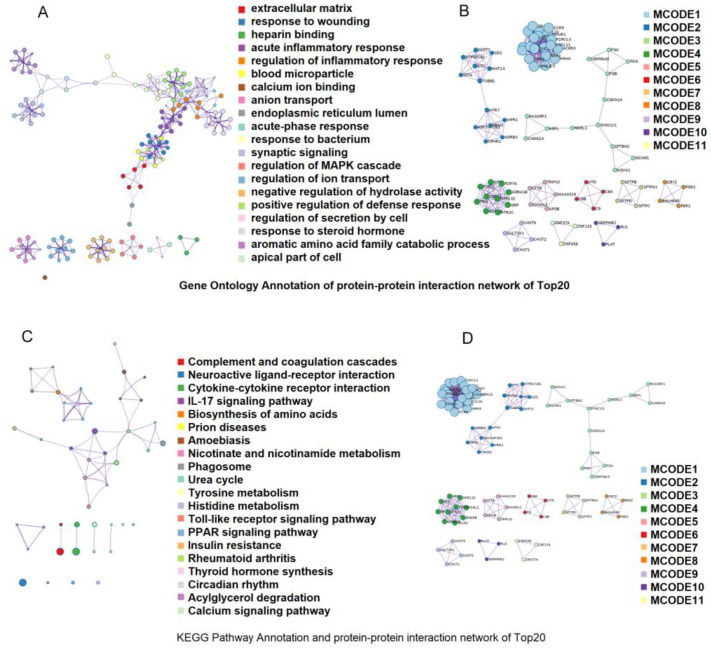
Function analysis of differentially expressed genes (DEGs) between the two groups based on the Gene Ontology and KEGG pathways. KEGG enrichment analysis of the differentially expressed genes [the top 20 most enriched pathway terms] (**A**); enriched KEGG pathway terms for the interaction network in MCODE components (**B**); enriched GO terms for the DEGs [the top 20 most enriched pathway terms] (**C**); and enriched GO terms for the interaction network in MCODE components (**D**).

Using the strict selection criteria of |log2 (fold change)| > 1 and q < 0.05, in order to compare the TP and YY, 1021 DEGs were screened. The 1021 differential genes in the TP vs. YY were mainly enriched on 1221 GO entries and 82 KEGG pathways. The GO terms for the top 20 predominately included the regulation of the MAPK cascade, blood microparticles, complement and coagulation cascades, and response to wounding (Figure 2A,B). The top 20 KEGG pathways mainly included the regulation of inflammatory responses, the IL-17 signaling pathway, the PPAR signaling pathway, insulin resistance, and thyroid hormone synthesis (Figure 2C,D).

### 3.3. RNA-seq Date Validation by RT-qPCR

**Figure 3:**
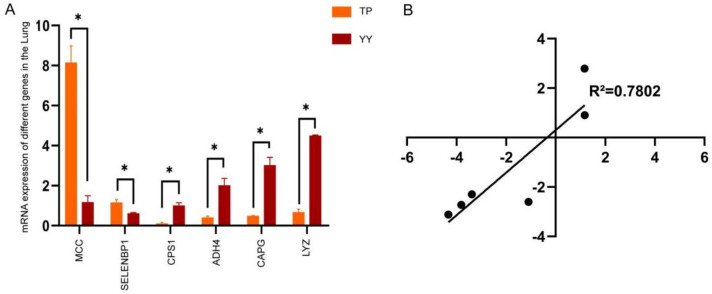
Validation of the RNA-Seq approach using RT-qPCR (**A**); transcriptome confirmation using real-time RT-qPCR [results represent means ± S.E.M.] (**B**); log2 fold changes in gene expression for RT-qPCR and RNA-Seq data, which are closely correlated (R = 0.7802; *p* = 0.0196), confirming the accuracy of the RNA-seq approach. * *p* < 0.05.

Validation of the RNA-sequence data was performed using six genes selected for analysis by RT-qPCR. The expression level of the *SELENBP1* gene in the TP was significantly higher than that in YY (*p* < 0.05), while the expression level of the *MCC* gene was significantly higher than that in YY (*p* < 0.01). The expression levels of the *CAPG* and *ADH4* genes in the TP were significantly lower (*p* < 0.05) than those in the YY. The expression levels of the *LYZ* and *CPS1* genes in the TP were significantly lower (*p* < 0.01) than those in the YY. Two genes were up-regulated and four were down-regulated. The quantitative results of the selected genes indicate their function and confirm the reliability of the omics data to a certain extent (Figure 3A). The correlation was evaluated in R using the RNA-seq log2 fold-change values and relative expression levels quantified by RT-q PCR. The correlation coefficient (R = 0.7802; *p* = 0.0196) revealed that gene expression levels were correlated in the data for the RT-qPCR and RNA-seq, which confirms the RNA-seq results (Figure 3B).

### 3.4. Protein Identification and Quantification

**Figure 4:**
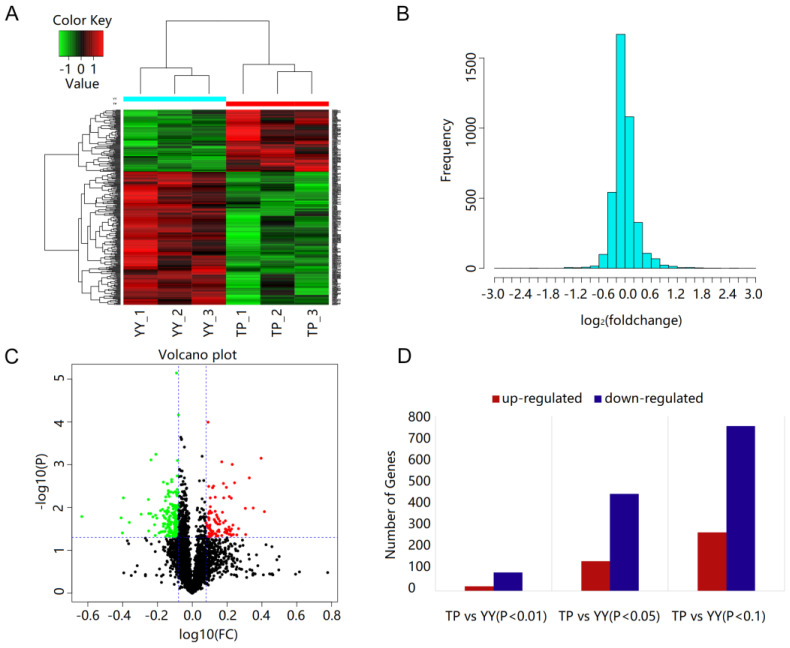
Cluster analysis of differentially expressed genes. Heatmap of differentially expressed proteins between two groups (**A**); distribution of identified proteins among the different molecular weight groups [in kDa] (**B**); volcano plot of differentially expressed proteins (**C**); and differentially expressed genes (**D**).

The total number of secondary spectra showed that a total of 72,991, and a total of 22,100 peptides matched in the proteome project. After filtering, a total of 4518 proteins were obtained under the condition of FDR < 0.01. A heatmap of all co-expressed proteins showed that the biological repeatability within each group was improved. There were differences in the proteome patterns of the TP and YY (Figure 4A). The clustering plots of all the expressed proteins showed that the biological repeatability of the two breeds was improved. The TP are typical plateau-adaptive animals, and significantly different from the YY. In terms of protein mass distributions, the proteins identified in the range of 10–70 kD account for approximately 64.94% (2934/4518; Figure 4B) of the total identified proteins. The volcano plot reflected the expressed uniproteins and showed that 88 proteins were up-regulated and 189 were down-regulated in the TP group when compared with the YY group (Figure 4C). The top 20 significantly up-regulated and down-regulated proteins in the two groups are listed in Table 2. Overall, 1023 (*p* < 0.1), 582 (*p* < 0.05), and 107 (*p* < 0.01) significant DEPs were identified between the TP and YY groups, including up-regulated and down-regulated UniGene IDs (Figure 4D).

### 3.5. Functional Annotation of DEPs

**Figure 5:**
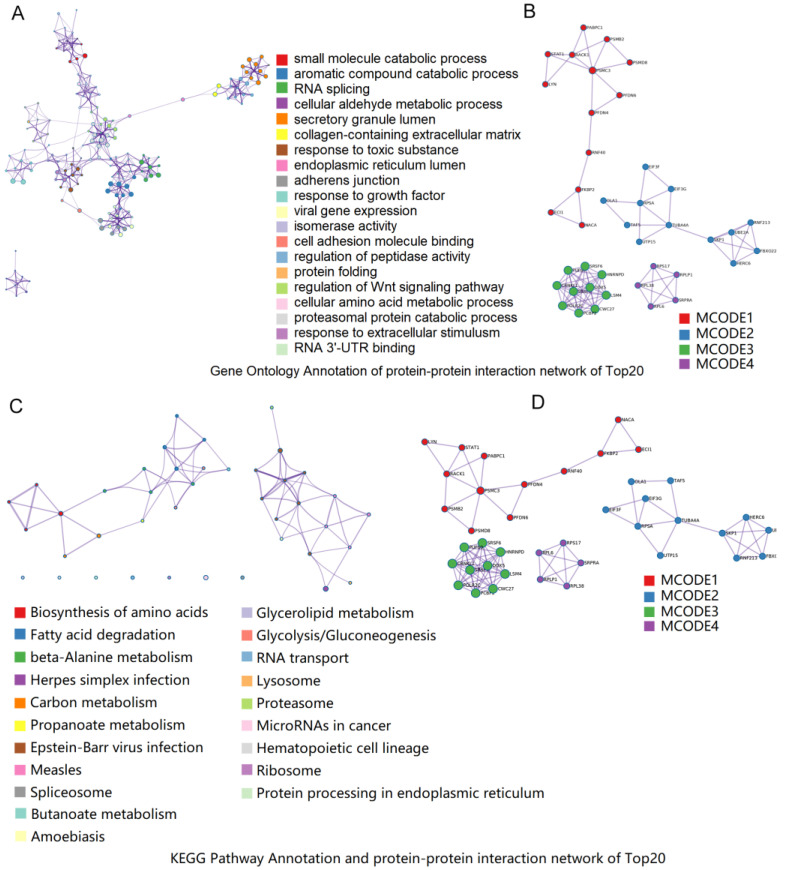
Function analysis of differentially expressed proteins (*DEPs*) between the two groups based on Gene Ontology and KEGG pathway analysis. KEGG enrichment analysis of the differentially expressed genes [the top 20 most enriched pathway terms] (**A**); enriched KEGG pathway terms for the protein–protein interaction network (**B**); enriched GO terms of the different expression genes [the top 20 most enriched pathway terms] (**C**); and enriched GO terms for the protein–protein interaction network (**D**).

According to the screening conditions of FC ≥ 1.8, FC ≤ 0.833, and *p* < 0.05 for the TP vs. YY, a total of 271 DEPs were identified. In the Metascape database, the 271 DEPs were significantly enriched in 902 GO entries and 45 KEGG pathways. The enriched GO entries mainly included the regulation of peptidase activity, regulation of Wnt signaling pathways, and responses to extracellular stimulus (Figure 5A,B). The enriched pathways mainly include MicroRNAs in cancer, hematopoietic cell lineage, and glycolysis/gluconeogenesis (Figure 5C,D).

### 3.6. Combined Analysis of DEGs in RNA-seq and DEPs in iTRAQ

**Figure 6:**
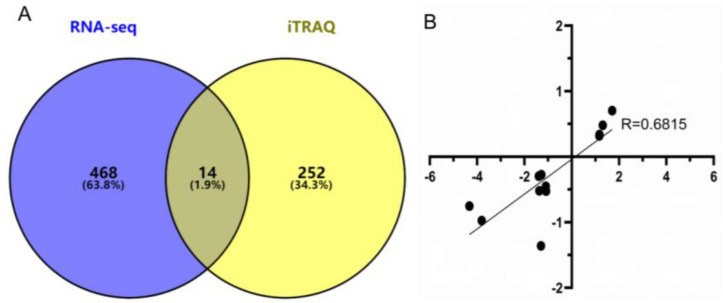
Bi-omics of the co-expressed genes/proteins. Transcriptome confirmation using real-time RT-qPCR [results represent means ± S.E.M.] (**A**); log2 fold changes in gene expression for the RNA-Seq and iTRAQ, which are closely correlated [R = 0.6815; *p* = 0.0005] (**B**).

The 1021 DEGs screened in the RNA-seq and the 271 *DEPs* screened in proteomics iTRAQ overlap with 22 genes; of these, 14 genes were annotated with the same trend (Figure 6A). A literature review and functional annotation of these 14 genes was performed. Among them, there were differentially expressed genes (*MCC*) on the Wnt signaling pathway: *ADH4* genes related to glycolysis/gluconeogenesis, ethanol oxidation, and quinone reductase activity NADPH regulation; *ASS1* genes related to mitochondrial function and *HIF-1* regulation; *CPS1* and *CAPG* [18,19,20,21], the *LYZ* genes associated with inflammatory responses and lysozyme activity; and the *SELENBP1* gene associated with selenium binding function, which serves as a marker for myocardial hypoxia [22]. The correlation was evaluated in R using the RNA-seq log2 fold-change values and relative expression levels quantified by iTRAQ. The correlation coefficient (R = 0.6815; *p* = 0.0005) revealed that gene expression was obtained by RT-qPCR and RNA-seq correlation and confirmed the correctness and reproducibility of the RNA-seq results (Figure 6B).

## 4. Discussion

Studies have shown that the hypoxic adaptation mechanism is a complex regulatory mechanism, and its occurrence and development are closely related to cardiopulmonary function [23]. In previous reports, plateau hypoxia adaptation-related pathways were analyzed in different types of pig lung tissue by combining RNA-seq and iTRAQ technology to identify relevant genes, including *A2M*, *COL3A1*, *CRYAB*, *DECR1*, and *PDLIM3* [13]. It has also been found that the immune mechanisms and anti-inflammatory effects of the pathway are also particularly important in hypoxic conditions [24]. The lungs are usually well-oxygenated organs and are sensitive to changes in oxygen content. The correlation between lung tissue and hypoxia adaptations has been widely observed. Controlled transcriptional responses are essential to optimize alveolar epithelial glucose metabolism, and thereby suppress lung inflammation during *ALI* [25]. TP, as a plateau-adapted breed, are better at adapting to plateau hypoxia than YY [26]. Many scholars have also conducted important comparisons between different breeds in order to investigate evolutionary patterns and energy metabolism [27] and the important functional roles of the genes between the different breeds [28]. The lung and cardiovascular tissues of TP have been shown to have unique advantages against the low oxygen of the plateau [29,30]. In this study, local TP and imported YY were selected as test subjects and their lung tissues were obtained for analyses. Hypoxia may have a certain impact on the lungs, and the expression of the key differentially expressed genes and proteins in lung tissue may also be related to hypoxia.

A combination of transcriptomics, proteomics, and dual expression omics analysis was used to screen and identify pathways and key genes related to hypoxia adaptations in the TP lung tissue. Transcriptome analysis revealed that gene expression is at the mRNA level and many complicated network mechanisms are involved in the expression of mRNA levels and the translation into protein [31,32]. The proteomics study identified the DEPs in the lung tissues of the two pig breeds at the protein level. The real-time fluorescence quantification of the key genes analyzed using the dual-omics analysis also showed the accuracy of transcriptomics data and the reliability of the identified genes. Consequently, the combination of the two-omics could provide more comprehensive and accurate gene expression information and could provide a reliable indicator for adaptations to plateau hypoxia.

A series of pathways and genes related to hypoxia adaptability from dual omics were screened. Liu [33] obtained the co-expression of genes by studying the molecular mechanisms of multiple myeloma (MM) and monoclonal gammopathy of undetermined significance. The complement and coagulation cascade- and *HIF-1*-related genes were significantly enriched in the network. Du [34] reported that complement and coagulation cascades play a particularly prominent role in adaptation to hypoxia; hypoxia-induced inflammation activation of JNK, and NF-κB signaling, lead to the phosphorylation of the *IRS-1* receptors by serine and impaired insulin signaling [35,36]. Hyperthyroidism can induce elevated plasma levels of fibronectin (FN by activating the HIF-1 pathway) up-regulation [37]; *HIF-1* responds to hypoxic-ischemic injury by inducing a type 3 deiodinase to reduce the regulation mechanism of local thyroid hormone signaling [38]. The cancer cell environment is often anoxic; under the hypoxia environment of lung cancer cells, changes in the Β-catenin’s position in the nucleus enhance Wnt signaling activities, thereby increasing the ability of lung cancer cells to induce chronic hypoxia [39,40,41,42].

Some studies have shown that the SLA protein family is closely related to stress resistance; TP have good stress resistance, which is consistent with the results of this study [43]. The expression level of the *SELENBP1* gene in the body is closely related to the duration of hypoxia and ischemia and is involved in myocardial hypoxia [44,45]. Earlier studies determined that MCC is closely related to the Wnt signaling pathway, a novel intracellular effector transducer, and to the regulation of cancer progression by the Wnt signaling pathway [46,47,48]. Hypoxia can cause pulmonary hypertension (HPH), and CAPG may promote or inhibit the proliferation of human pulmonary artery smooth muscle cells (PASMCs). It grows to participate in the mechanism of pulmonary vascular remodeling in HPH rats, and the expression of *CAPG* is also induced by hypoxia [49,50]. The arginine succinate synthetase 1 (*ASS1*) is an arginine (Arg) biosynthesis process (key enzyme in HIF-1) that controls the silencing of *ASS1* and starves Arg, thereby inhibiting the growth of Arg vegetative tumor cells [19,51]. The alcohol dehydrogenase 4 (*ADH4*) is involved in ethanol oxidation and glycolysis and is related to hypoxia adaptation pathways [52,53]. During physiological changes when adapting to a hypoxic environment, the innate immune response and other test results for the expression level of the *LYZ* gene increased with prolonged hypoxia time [54]. Carbamoyl phosphate synthetase-1 (CPS; key mitochondrial rate-limiting enzyme in urea cycle) in a hypoxic environment of tumor cells can reduce cell growth and prevent the production of metabolites in the nucleic acid biosynthetic pathway [55,56].

It is worth mentioning that an interesting phenomenon was found in this study. Some genes that have been shown to be related to hypoxia stress response are up-regulated in the RNA-seq omics of this study, but down-regulated in the proteomics, including *CRYAB* [57] and *HIF-1A* [58], among others. We speculate that this may be due to a certain modification during RNA translation into protein, which may also be due to the interaction with other genes or proteins in the regulatory role. The specific regulatory mechanism needs further study.

## 5. Conclusions

In total, approximately 1021 DEGs were identified in the lung tissues of the TP and YY pigs using RNA-seq, and 271 DEPs were obtained using iTRAQ. Transcriptome, proteome, and dual expression levels for six key candidate genes (*SELENBP1*, *MCC*, *CAPG*, *ADH4*, *LYZ*, and *CPS1*) were screened from the lung tissues, and they were found to be predominantly involved in mitochondrial function, blood particle regulation, glycolysis, ethanol oxidation, and the Wnt signaling pathway, as well as other related hypoxia-adaptive regulatory mechanisms.

## Figures and Tables

**Table 1:** Detail information for the top 20 differentially expressed genes.

Gene ID	Gene Name	Log2 Fold Change	*p*-Value	Up/Down
ENSSSCG00000009738	*GALNT9*	7.689227	1.83 × 10^−^^6^	up
ENSSSCG00000033254	*IFN-ALPHA-13*	6.983059	1.02 × 10^−6^	
ENSSSCG00000007859	*UMOD*	6.040727	0.009784	
ENSSSCG00000033610	*ZG16B*	6.015486	0.024330	
ENSSSCG00000037533	*HIST2H2AB*	5.656798	0.001126	
ENSSSCG00000029516	*SLC22A8*	5.639100	0.020157	
ENSSSCG00000034995	*RF00017*	5.098178	0.035442	
ENSSSCG00000037202	*CACNG4*	4.860514	0.013594	
ENSSSCG00000037225	*RPRM*	4.856914	0.016825	
ENSSSCG00000008741	*FGFBP1*	4.832671	0.000081	
ENSSSCG00000037535	*SLC34A1*	4.729766	0.013466	
ENSSSCG00000037300	*GRP*	4.443211	0.005612	
ENSSSCG00000035689	*NEXMIF*	4.430799	0.043955	
ENSSSCG00000001906	*CYP1A1*	4.426375	0.000505	
ENSSSCG00000003066	*IRGC*	4.404553	0.047451	
ENSSSCG00000033193	*TPO*	4.400171	0.016735	
ENSSSCG00000037534	*OPCML*	4.233214	4.60 × 10^−^^16^	
ENSSSCG00000001613	*TREML1*	4.174212	0.003859	
ENSSSCG00000028695	*TMSB15A*	4.032314	3.94 × 10^−^^14^	
ENSSSCG00000022140	*TMPRSS11E*	4.005328	0.028356	
ENSSSCG00000040910	*APOH*	−4.214353	0.027279	down
ENSSSCG00000021767	*TDH*	−4.268103	0.001937	
ENSSSCG00000002479	*SERPINA11*	−4.286988	0.006838	
ENSSSCG00000012711	*F9*	−4.323437	0.026465	
ENSSSCG00000016159	*CPS1*	−4.326881	0.000381	
ENSSSCG00000012517	*TMSB15B*	−4.341481	5.88 × 10^−^^7^	
ENSSSCG00000016856	*C9*	−4.466406	0.037223	
ENSSSCG00000010431	*A1CF*	−4.487968	0.017588	
ENSSSCG00000003835	*C8A*	−4.594111	0.000224	
ENSSSCG00000020680	*CLDN14*	−4.631278	0.006969	
ENSSSCG00000008998	*FGA*	−4.729940	0.002310	
ENSSSCG00000036158	*TRAM1L1*	−4.734145	0.003310	
ENSSSCG00000037547	*SLC17A3*	−4.893193	0.000000	
ENSSSCG00000006248	*MOS*	−5.303923	0.006916	
ENSSSCG00000002983	*LGALS13*	−5.369302	0.000084	
ENSSSCG00000034429	*PLA2G5*	−5.410249	0.010311	
ENSSSCG00000029449	*PRG4*	−5.667579	4.54 × 10^−^^20^	
ENSSSCG00000016315	*SPP2*	−6.047557	0.000038	
ENSSSCG00000008214	*FABP1*	−6.452786	0.000014	
ENSSSCG00000037268	*APCS*	−7.280566	2.05 × 10^−7^	

If the genes expression level is higher in Tibetan pigs (TP) than in Yorkshire pigs (YY), it is up-regulated, otherwise it is down-regulated.

**Table 2:** Detail information for the top 20 differentially expressed proteins.

Accession	Gene Name	MW [kDa]	FC	*p*-Value	Log2FC	
A0A286ZWS8	*COL2A1*	141.6	4.119454	0.321115	2.042453	up
F8WSC1	*SLA-1*	40	3.918033	0.357562	1.970129	
V9PR54	*SLA-1*	40.4	3.158697	0.138104	1.659330	
A0A287AEL2	*KRT14*	56	3.135079	0.288655	1.648502	
A0A287ATD0	*KRT75*	58.7	3.059540	0.166403	1.613315	
I3L8B2	*COL9A2*	65.1	2.900520	0.371928	1.536312	
A0A287B863	*ACAN*	251.9	2.875969	0.352952	1.524048	
F1S571	*COL11A1*	147.1	2.861647	0.081541	1.516846	
F1REZ1	*HAPLN1*	40.2	2.843049	0.359189	1.507439	
A0A286ZI25	*PARP14*	200.9	2.780718	0.354096	1.475458	
F2Z501	*TMED2*	21.7	2.649027	0.073811	1.405462	
F1S0J1	*C4BPA*	22.7	2.597122	0.012400	1.376914	
F1SCU3	*MATN3*	52.7	2.556017	0.376462	1.353897	
F1RXG1	*KRT27*	49.7	2.384095	0.227473	1.253442	
A5A758	*KRT1*	65.2	2.333889	0.126611	1.222736	
A0A287A461	*CHAD*	40.6	2.294893	0.355746	1.198427	
F1S7K4	*PLIN4*	158.7	2.237992	0.010325	1.162205	
I3LDS3	*KRT10*	58.9	2.234501	0.340817	1.159953	
I3L5Q7	*MATN1*	53.9	2.227542	0.361420	1.155452	
A7J149	*BPIFB1*	51.9	2.227004	0.377700	1.155104	
F1SIT7	*RPLP1*	11.5	0.576307	0.013941	−0.795091	down
F1RW28	*HSD17B13*	33.3	0.561833	0.013894	−0.831787	
F1RL41	*UPB1*	42.9	0.560874	0.035026	−0.834251	
A0A287BN06	*PZP*	158.1	0.560354	0.006529	−0.835590	
B5L2L8	*SLA-DQA*	9.5	0.546931	0.058481	−0.870568	
A0A0A7BZH1	*SLA-DQB1*	29.5	0.538462	0.365487	−0.893085	
L7UWL8	*SLA-2*	20.7	0.538067	0.396005	−0.894142	
A0A2C9F382	*FABP1*	16.5	0.537673	0.051562	−0.895199	
A0A2C9F343	*LYZ*	16.5	0.508926	0.014242	−0.974471	
Q8HX61	*SLA-B*	40.6	0.482827	0.230141	−1.050422	
B6DU23	*SLA-DRB1*	10.8	0.470833	0.319788	−1.086712	
B6ICW6	*SLA-2*	40.5	0.445435	0.385493	−1.166714	
D3GIN8	*SLA-2*	38.8	0.435303	0.022197	−1.199910	
K9J6H8	*A2M*	163.9	0.430956	0.069842	−1.214386	
A0A1L1YNR3	*FASN*	93.4	0.423150	0.060034	−1.240759	
T2HGI4	*SLA-1*	35	0.404494	0.333977	−1.305808	
D3GIP1	*SLA-3*	40.4	0.402103	0.005976	−1.314364	
I3LLB7	*PIK3R1*	83.5	0.396648	0.039119	−1.334069	
F1SER3	*SFTPA1*	26.5	0.389442	0.017424	−1.360520	
A0A1C9J6L2	*SLA-1*	40.1	0.231828	0.016153	−2.108876	

If the protein expression level is higher in Tibetan pigs (TP) than in Yorkshire pigs (YY), it is up-regulated, otherwise it is down-regulated.

## Data Availability

Raw data are available upon request from the corresponding authors.
